# Applying implementation frameworks to the clinical trial context

**DOI:** 10.1186/s43058-022-00355-6

**Published:** 2022-10-10

**Authors:** Kristian D. Stensland, Anne E. Sales, Laura J. Damschroder, Ted A. Skolarus

**Affiliations:** 1 Dow Division of Health Services Research, Department of Urology, University of Michigan, NCRC Building 16, 100S-12, 2800 Plymouth Road, Ann Arbor, MI 48109 USA; 2 Department of Learning Health Sciences, University of Michigan, Ann Arbor, MI USA; 3 Center for Clinical Management Research, VA Ann Arbor Healthcare System, Ann Arbor, MI USA; 4 Sinclair School of Nursing, University of Missouri, Columbia, MO USA; 5 Department of Family and Community Medicine, University of Missouri School of Medicine, Columbia, MO USA

**Keywords:** Implementation outcomes, Determinants, Implementation research logic model, Consolidated framework for implementation research, Implementation mapping, Clinical trials

## Abstract

**Background:**

Clinical trials advance science, benefit society, and provide optimal care to individuals with some conditions, such as cancer. However, clinical trials often fail to reach their endpoints, and low participant enrollment remains a critical problem with trial conduct. In these ways, clinical trials can be considered beneficial evidence-based practices suffering from poor implementation. Prior approaches to improving trials have had difficulties with reproducibility and limited impact, perhaps due to the lack of an underlying trial improvement framework. For these reasons, we propose adapting implementation science frameworks to the clinical trial context to improve the implementation of clinical trials.

**Main text:**

We adapted an outcomes framework (Proctor’s Implementation Outcomes Framework) and a determinants framework (the Consolidated Framework for Implementation Research) to the trial context. We linked these frameworks to ERIC-based improvement strategies and present an inferential process model for identifying and selecting trial improvement strategies based on the Implementation Research Logic Model. We describe example applications of the framework components to the trial context and present a worked example of our model applied to a trial with poor enrollment. We then consider the implications of this approach on improving existing trials, the design of future trials, and assessing trial improvement interventions. Additionally, we consider the use of implementation science in the clinical trial context, and how clinical trials can be “test cases” for implementation research.

**Conclusions:**

Clinical trials can be considered beneficial evidence-based interventions suffering from poor implementation. Adapting implementation science approaches to the clinical trial context can provide frameworks for contextual assessment, outcome measurement, targeted interventions, and a shared vocabulary for clinical trial improvement. Additionally, exploring implementation frameworks in the trial context can advance the science of implementation through both “test cases” and providing fertile ground for implementation intervention design and testing.

Contributions to the literature

- Clinical trials advance science and benefit society and individuals, but suffer from poor implementation.
- Existing methods to improve clinical trial conduct have had limited effectiveness, maybe due to the lack of a unifying framework.
- We address this knowledge gap by adapting implementation science frameworks to the clinical trial context.
- We provide a worked example of our proposed model applied to an example of poor trial enrollment to demonstrate the utility of this targeted approach.

## Introduction

Clinical trials are critical components of research and healthcare infrastructure with hundreds of thousands of participants enrolled and billions of dollars invested annually [1]. In addition to advancing science, trials can ensure adequate and even improved care for patients through a “protocol effect” by building infrastructure and disseminating knowledge about the standard of care treatments [2]. In fact, for some conditions, such as cancer, many consider enrollment in a clinical trial to be the best possible management [3]. Despite these advantages and investments, clinical trials frequently fail to reach their primary endpoints, commonly do not meet enrollment goals, and often take longer than anticipated to enroll and complete [4–7]. In these ways, clinical trials can be considered complex evidence-based interventions with significant benefits to both individuals and society although suffering from poor implementation [8]. The clinical trials system could benefit from implementation science approaches to address this evidence-to-practice gap.

While there have been prior attempts to improve clinical trials, interventions have generally not been reproduced, led to sustainable improvement, or been grounded in theory, limiting generalizability [9]. In other words, clinical trials have suffered from poor implementation and limited improvement efforts similar to other complex evidence-based interventions. By considering clinical trials as complex interventions with poor implementation, the existing knowledgebase for assessing and addressing poor implementation of other complex interventions (e.g., smoking cessation, cancer screening) can be applied to the clinical trial context [10, 11]. Building out of existing implementation work rather than establishing entirely de novo techniques for clinical trial implementation can facilitate the application of evidence-based strategies and frameworks to the trial context. Adapting implementation frameworks to the clinical trial context can also advance science via new application of a shared vocabulary and improvement models to generate new knowledge. While some frameworks have been applied to aspects of clinical trials, a global consideration of trials as complex interventions through an implementation science lens could significantly advance both the science and practice of clinical trials and the field of implementation science [12, 13].

For these reasons, we applied implementation science frameworks to the clinical trial context as a worked example of the potential opportunity to advance the practice and science of clinical trials and implementation. Specifically, we adapted Proctor’s implementation outcomes framework (IOF) to develop corresponding clinical trial implementation outcomes informing external validity (e.g., acceptability) in addition to internal validity (e.g., reproducibility). Next, we used the Consolidated Framework for Implementation Research (CFIR) to define context and determinants specific to clinical trial implementation. We then mapped contextual determinants to possible Expert Recommendations for Implementing Change (ERIC) strategies to guide implementation interventions. Finally, we used the implementation research logic model (IRLM) as a rigorous tool to facilitate specification, reproducibility, and testable causal mechanisms of the interventions on implementation and clinical trial outcomes [14–17]. Through this worked example applying implementation science frameworks and approaches to the clinical trial context, we hope to bolster a foundation and build capacity for rigorous, evidence-based clinical trial improvement.

## Considering clinical trial implementation outcomes

**Fig. 1:**
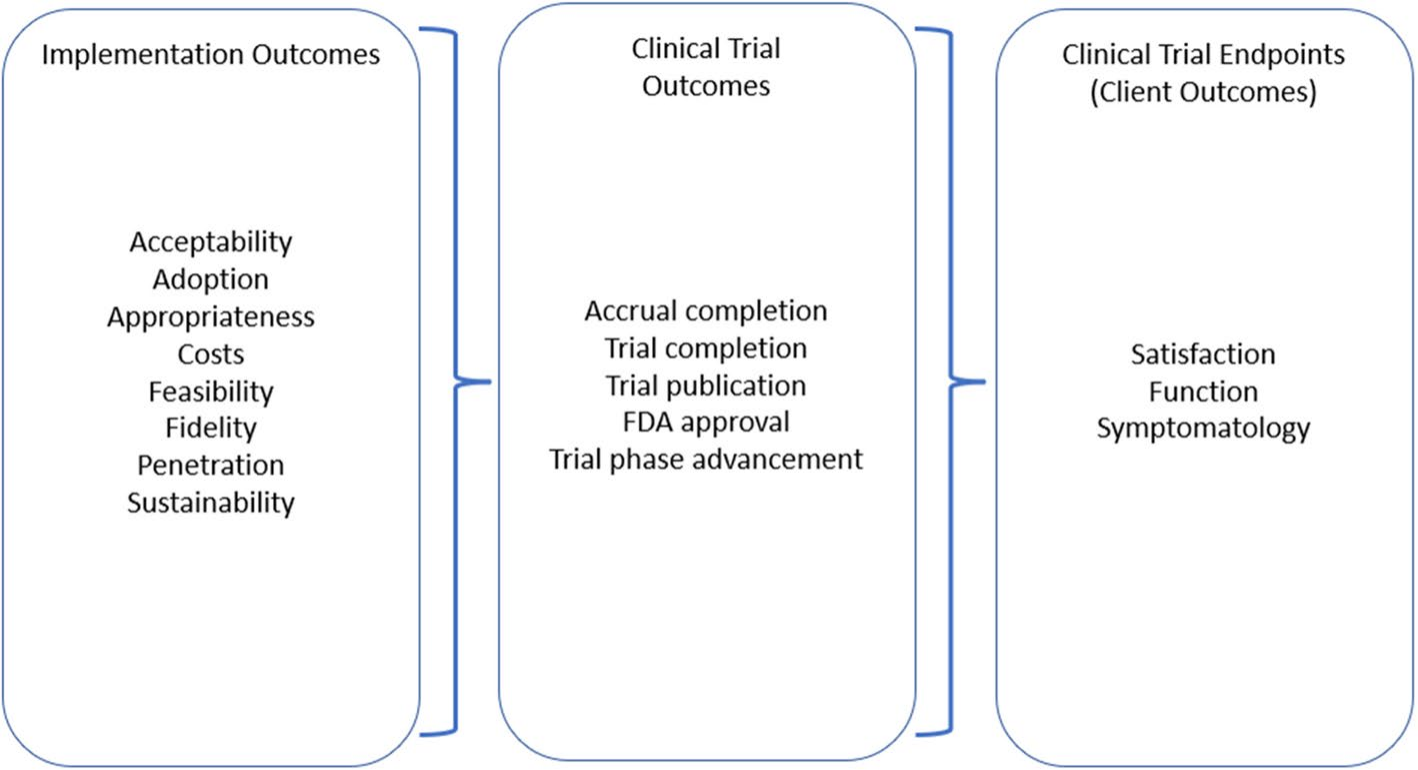
Implementation, service, and client outcomes adapted to the clinical trial context

In the context of clinical trials, “outcomes” generally refer to the primary or secondary outcomes of the trial itself, such as overall survival for a cancer treatment trial. To avoid confusion within this paper, we will refer to these as “endpoints” rather than outcomes. While consideration of trials normally focuses on reaching these endpoints, trials must meet other preconditions to facilitate this objective. For example, a clinical trial must enroll and retain enough participants to answer the trial’s question. However, preconditions and the best ways to meet them to achieve trial endpoints remain poorly defined. Similar to a clinical setting, where client outcomes (e.g., satisfaction, symptomatology) and service outcomes (e.g., effectiveness, safety) are preceded by implementation outcomes (e.g., acceptability, adoption), we suggest clinical trial endpoints are based on certain necessary preconditions well suited as implementation outcomes [15]. As illustrated in Fig. 1, the endpoints of clinical trials correlate to client-side outcomes in Proctor’s implementation outcomes framework (IOF), aligning with successful attainment of intermediate service outcomes and preconditioned on implementation outcomes to reach ultimate client-side success, i.e., reaching clinical trial endpoints and improving patient satisfaction, function, and/or symptoms.

Proctor et al. described eight implementation outcomes in the IOF: acceptability, adoption, appropriateness, feasibility, fidelity, implementation cost, penetration, and sustainability [15]. Each of these implementation outcomes aligns with important considerations for clinical trial design and implementation. As shown in Table 1, our worked example proposes measures of each of these outcomes in the trial context. Indeed, some measures are currently considered in trial design and analysis, such as feasibility and fidelity, though these terms are not always used. For example, fidelity to a trial’s intervention is sometimes referred to as “contamination” or “crossover.” Reframing these existing concepts within the implementation science context has the potential to realign the direction of improvement efforts towards existing, evidence-based implementation strategies.

**Table 1:** Implementation outcomes framework applied to the clinical trial context

Proctor’s implementation outcome	Example in the clinical trial context
Acceptability	Perceived existence of equipoise between intervention arms
	Anticipated or possible benefit to a participant over existing options
	Acceptable anticipated side effect profile
	Reasonable participant logistics (e.g., number of clinic visits, distance traveled to the trial site)
	Reasonable additional clinical burden (e.g., minimal additional biopsies or other invasive procedures)
	Additional direct time and financial cost to participants is acceptable to participants
Adoption	Proportion of providers offering clinical trials to patients
Appropriateness	Question is amenable to a clinical trial
	Trial design is appropriate for the trial question
Feasibility	Possible to meet enrollment goals
	Timeline for enrollment and completion is reasonable
	Anticipated effect size is reasonable
Fidelity	Amount of intervention group crossover
	Adherence to trial protocol including follow-up
Implementation cost	Cost of trial administration
	Cost of trial intervention vs. standard of care (during trial)
	Cost of additional trial staff required
	Cost of additional study components (surveys, labs, scans, biopsies)
Penetration	Proportion of eligible patients being offered trial
	Proportion of eligible patients offered trial who enroll in the trial
	Proportion of patients in the global population represented by trial eligibility criteria
Sustainability	Maintenance of accrual rates after trial opens
	Sustained physician interest (i.e., physicians continue offering trial to patients throughout the trial period)
	Sustained participant interest throughout the trial period
	Continued provision of standard of care after the trial concludes

Additionally, this may shift focus from retrospective analyses of completed clinical trials to prospective considerations and testable interventions during trial design and implementation, encouraging more efficient clinical trial design. The outcomes serve as both a measure of trial implementation success and as a checklist to encourage consideration of multifaceted factors in trial design and trial site recruitment phases. Shifting assessment of trial success from historical endpoints (i.e., waiting for interim enrollment and endpoint analysis) to consideration of implementation outcomes at the time of trial design and in the early stages of trial implementation could move important assessments up front, saving time and resources for participants, trialists, and sponsors. In other words, implementation outcomes in the trial context may not just measure the implementation success of trials, their use per se may also improve trial success.

Finally, clinical trial implementation outcomes could also be used as endpoints in trial improvement studies. For example, a trial of an intervention to improve clinical trial enrollment could benefit from defining the primary endpoints as adoption (the number of providers offering at least one patient enrollment on a clinical trial) and penetration (the proportion of eligible patients offered enrollment on a clinical trial). This would also encourage assessing the factors leading to successful implementation, such as acceptability to clinician trialists. By consistently defining and applying these measures, the body of evidence for trial improvement interventions could be more easily generalized, compared, and consolidated into recommendations for optimal, evidence-based trial conduct.

### Clinical trials as test cases of implementation outcomes

In addition to trials benefiting from the use of implementation outcomes, the trial context can contribute to implementation science as a helpful setting for exploring nuanced outcomes and the relationships between them. Indeed, multi-center trials have the advantage of tracking what happens across different settings as every center implements trials somewhat differently although rarely is trial implementation the same across centers raising implementation and causality questions. Next, we provide more concrete examples of implementation outcomes and concepts in the trial context to help clarify these otherwise abstract concepts.

For example, there can be considerable overlap in the implementation outcomes of *appropriateness* (perceived fit of innovation) and *acceptability* (perceived palatability of innovation), raising the question of why distinguishing between them is important [15]. For a clinical trial, the distinction between these concepts is both clear and highly impactful. A trial is *appropriate* if a given clinical trial design is the correct way to answer a question. For example, testing an intervention in schools by randomizing individual students to two interventions would not be *appropriate*; a better design would be a cluster randomized trial. In contrast, the *acceptability* of a trial reflects the palatability of selected interventions to potential participants or providers. A trial may have low *acceptability* because one of the interventions is highly toxic, or because there are many required return visits making the trial logistically challenging for participants. A trial may also have low *acceptability* to providers due to perceived superiority of one intervention (i.e., lack of perceived equipoise in the trial). This may be an explanation for the difficulty in enrolling participants with cancer in a radiation therapy versus surgery trial (*appropriate* design), as surgeons may not be willing to randomize patients to non-operative care and radiation oncologists may be unwilling to randomize patients to surgery (*unacceptable* to providers) [18]. Similarly, though a trial of an antibiotic versus placebo for a blood infection may be an *appropriate* design to demonstrate the effectiveness of the antibiotic, this would be considered highly unethical and thus not an *acceptable* design.

Non-trial settings may also have poorly characterized relationships between implementation outcomes (e.g., relationships between acceptability and appropriateness) [15]. In this regard, the trial context comprises a contained setting facilitating the exploration of explicit implementation outcome trade-offs. In non-trial interventions, a trade-off may only be about the *implementation cost*, i.e., if more resources are available, problems with *feasibility* or *penetration* may be easily addressed. However, other trial implementation outcome trade-offs are more complex. To make a trial more *feasible*, trial eligibility criteria could be expanded, but this may require larger sample sizes to meet the efficacy endpoint of the trial. As a result, the trial may take longer to enroll, and *sustainability* may suffer as providers lose interest in the trial resulting in waning *adoption* and *penetration*. Alternatively, decreasing the number of follow-up visits in a trial may enhance *acceptability* to participants and decrease *implementation cost*, but the trial results may be less useful or reliable, reflecting a lower *appropriateness* of the trial. While these tradeoffs likely exist in other implementation settings, they are often not as visible or immediate as in clinical trials. Studying these relationships and tradeoffs between outcomes within the clinical trials setting could allow for more rapid study and development of frameworks that could then be highlighted and addressed in the implementation of other evidence-based practices and the field more broadly.

## Applying a determinants framework to the trial context

Optimizing trial success through these implementation outcomes requires the identification of implementation determinants. This can aid in identifying barriers and facilitators to trial success and lead to selecting trial improvement interventions in a rigorous, theory-based way to enable testable causal hypotheses advancing implementation science.

The Consolidated Framework for Implementation Research (CFIR) is a robust, frequently used determinants framework [16]. Its 37 constructs across 5 domains represent key components of clinical trials as complex interventions likely influencing implementation success. Our suggested adaptation of each construct is shown in Table 2. The overarching CFIR domains containing these constructs reflect multiple levels of trial implementation and connect to the adapted Proctor outcomes described above. We propose considering these domains as follows.

**Table 2:** Adaptation of the CFIR domains and constructs to the clinical trial context

Construct		Construct description	Example of adaptation to clinical trial context
**I. Intervention characteristics**			
A	Intervention source	The group that developed and/or visibly sponsored use of the innovation is reputable, credible, and/or trustable	Perception of providers about whether the trial protocol, or experimental intervention, was developed at the provider’s institution Perception of patients about whether patients were involved in the design of a trial
B	Evidence strength and quality	Stakeholders’ perceptions of the quality and validity of evidence supporting the belief that the intervention will have desired outcomes	Providers’ or patients’ perception of the quality and validity of existing evidence for the experimental intervention. This may include data from pilot trials or trials of earlier phases. This contributes to the perceived equipoise of a trial Providers’ or patients’ perception of the quality and validity of existing evidence for the comparison arm, i.e., “standard of care”
C	Relative advantage	Stakeholders’ perception of the advantage of implementing the intervention versus an alternative solution	Providers’ or patients’ perception of the experimental intervention versus the comparison (e.g., standard of care) arm or alternative intervention. This contributes to the perceived equipoise of a trial
D	Adaptability	The degree to which an intervention can be adapted, tailored, refined, or reinvented to meet local needs	The degree to which a trial protocol can be adapted to local needs or fit into local practice
E	Trialability	The ability to test the intervention on a small scale in the organization, and to be able to reverse course (undo implementation) if warranted	The ability to switch to standard of care or other treatments if desired The ability to pilot on a smaller scale before launching a larger trial
F	Complexity	Perceived difficulty of implementation, reflected by duration, scope, radicalness, disruptiveness, centrality, intricacy, and number of steps required to implement	Perceived logistical complexity (number of return visits, labs) and invasiveness (extra procedures including biopsies), and duration of follow-up required for trial participation
G	Design quality and packaging	Perceived excellence in how the intervention is bundled, presented, and assembled	Perceived excellence in the trial materials and advertising
H	Cost	Costs of the intervention and costs associated with implementing the intervention including investment, supply, and opportunity costs	Cost of trial to patients, institution, and/or insurers Impact of trial participation on provider reimbursement
**II. Outer setting**			
A	Patient needs and resources	The extent to which patient needs, as well as barriers and facilitators to meet those needs, are accurately known and prioritized by the organization	The extent to which a trial reflects local needs, for example, regional disease prevalence The extent to which desires of patients/trial participants (e.g., measuring endpoints, desired toxicity cut points, ability to participate) are known and used in trial design
B	Cosmopolitanism	The degree to which an organization is networked with other external organizations	The degree to which the trial site organization is networked with other external organizations, such as cancer cooperative groups (e.g., SWOG) or other research networks
C	Peer pressure	Mimetic or competitive pressure to implement an intervention; typically because most or other key peer or competing organizations have already implemented or are in a bid for a competitive edge	Competitive pressure to run and enroll participants in trials, particularly to compete with other investigators or institutions for NCI designations and institutional rankings
D	External policy and incentives	A broad construct that includes external strategies to spread interventions, including policy and regulations (governmental or other central entity), external mandates, recommendations and guidelines, pay-for-performance, collaboratives, and public or benchmark reporting	Government and insurance company reimbursement for clinical trials Financial incentives from cooperative and government grants for trial accrual
**III. Inner setting**			
A	Structural characteristics	The social architecture, age, maturity, and size of an organization	The history of clinical trials at an institution, including past performance and influence on other centers The social architecture, age, maturity, and size of an organization
B	Networks and communications	The nature and quality of webs of social networks and the nature and quality of formal and informal communications within an organization	The nature and quality of social networks and communications within an organization, such as frequency of division and department meetings, tumor boards, and other communication networks The physical proximity of clinical and clinical research spaces (i.e., opportunities for informal conversation about trials)
C	Culture	Norms, values, and basic assumptions of a given organization	Culture of research and trials within an institution and department
D	Implementation climate	The absorptive capacity for change, shared receptivity of involved individuals to an intervention, and the extent to which use of that intervention will be rewarded, supported, and expected within their organization	Institutional and departmental support/incentives for trial participation, and available resources for trial implementation Institutional and departmental recognition for trial participation
1	Tension for change	The degree to which stakeholders perceive the current situation as intolerable or needing change	The degree to which providers perceive there to be a need for a trial (e.g., poor health outcomes for a condition)
2	Compatibility	The degree of tangible fit between meaning and values attached to the intervention by involved individuals, how those align with individuals’ own norms, values, and perceived risks and needs, and how the intervention fits with existing workflows and systems	The degree of fit between a trial and the provider’s norms, values, and perceived risks and needs (i.e., the provider’s practice) The degree of fit between a trial and the participant’s norms, values, and perceived risks and needs
3	Relative priority	Individuals’ shared perception of the importance of the implementation within the organization	Providers’ shared perception of the importance of the trial within the organization Providers’ shared perception of the importance of conducting any clinical trial
4	Organizational incentives and rewards	Extrinsic incentives such as goal-sharing awards, performance reviews, promotions, and raises in salary, and less tangible incentives such as increased stature or respect	Provider incentives such as career advancement, salary support, and increased reputation for trial participation
5	Goals and feedback	The degree to which goals are clearly communicated, acted upon, and fed back to staff, and alignment of that feedback with goals	The degree to which trial goals (e.g., enrollment goals) are clearly communicated, acted upon, and fed back to staff and aligned with organizational goals
6	Learning climate	A climate in which: a) leaders express their own fallibility and need for team members’ assistance and input; b) team members feel that they are essential, valued, and knowledgeable partners in the change process; c) individuals feel psychologically safe to try new methods; and d) there is sufficient time and space for reflective thinking and evaluation	A climate in which: a) trial leaders (e.g., trialists and institutional leaders) express their own fallibility and need for team members’ assistance and input; b) trial team members feel that they are essential, valued, and knowledgeable partners in the change process; c) individual trialists and providers feel psychologically safe to try new methods; and d) there is sufficient time and space for reflective thinking and evaluation
E	Readiness for implementation	Tangible and immediate indicators of organizational commitment to its decision to implement an intervention	Tangible indicators of organization commitment to the trial
1	Leadership engagement	Commitment, involvement, and accountability of leaders and managers with the implementation	Commitment, involvement, and accountability of institutional leaders with the trial
2	Available resources	The level of resources dedicated for implementation and on-going operations, including money, training, education, physical space, and time	The level of available resources for trials, including staffing (e.g., research nurse, administrative staff), logistics (e.g., IRB and protocol support), time, and contributed resources (e.g., provision of space)
3	Access to knowledge and information	Ease of access to digestible information and knowledge about the intervention and how to incorporate it into work tasks	Availability of clinical trial training including design, proposals, statistics, logistics and trial-specific training Availability of trial information, such as which trials are open, eligibility criteria, and protocols
**IV. Characteristics of individuals**			
A	Knowledge and beliefs about the intervention	Individuals’ attitudes toward and value placed on the intervention as well as familiarity with facts, truths, and principles related to the intervention	Providers’ and patients’ attitude toward and value placed on clinical trials and research as well as familiarity with the science of clinical trials, considerations of equipoise, and trial ethics
B	Self-efficacy	Individual belief in their own capabilities to execute courses of action to achieve implementation goals	Belief of potential participants and involved clinicians (recruiters) that trial will complete and/or have an impact
C	Individual stage of change	Characterization of the phase an individual is in, as he or she progresses toward skilled, enthusiastic, and sustained use of the intervention	Individual trialist or provider willingness to fulfill trial role with skill and enthusiasm
D	Individual identification with organization	A broad construct related to how individuals perceive the organization, and their relationship and degree of commitment with that organization	Strength of commitment of investigator and trial to sponsoring institution
E	Other personal attributes	A broad construct to include other personal traits such as tolerance of ambiguity, intellectual ability, motivation, values, competence, capacity, and learning style	Characteristics of those involved with implementing the trial (i.e., research team, providers), including risk tolerance, acceptance of being the subject of an experiment, personal attitudes towards science, trust in authority, etc
**V. Process**			
A	Planning	The degree to which a scheme or method of behavior and tasks for implementing an intervention are developed in advance, and the quality of those schemes or methods	The degree to which trial recruitment plans are developed in advance, including feasibility planning The degree to which deviations to trial protocols (e.g., changes in standard of care) are anticipated and adaptations developed in advance
B	Engaging	Attracting and involving appropriate individuals in the implementation and use of the intervention through a combined strategy of social marketing, education, role modeling, training, and other similar activities	Attracting and involving appropriate providers and patients in trials through a combined strategy of social marketing, education, role modeling, training, and other similar activities
1	Opinion leaders	Individuals in an organization who have formal or informal influence on the attitudes and beliefs of their colleagues with respect to implementing the intervention	Key leaders in cancer center, trials support unit, and within a department/division
2	Formally appointed internal implementation leaders	Individuals from within the organization who have been formally appointed with responsibility for implementing an intervention as coordinator, project manager, team leader, or other similar roles	Individuals from within the organization who have been formally appointed with responsibility for leading conduct of a trial, including trial principal investigator (PI), trial site PI, trial coordinator, or trials team coordinator
3	Champions	“Individuals who dedicate themselves to supporting, marketing, and ‘driving through’ an [implementation]”, overcoming indifference or resistance that the intervention may provoke in an organization	Individuals who dedicate themselves to supporting, marketing, and “driving through” a trial, including advertising a trial to providers and patients, advocating for trials at meetings such as tumor boards, and pushing trial materials through review boards
4	External change agents	Individuals who are affiliated with an outside entity who formally influence or facilitate intervention decisions in a desirable direction	Other stakeholders who guide trial conduct such as trial cooperative group members, trial PIs from other institutions for multisite trials, patient advocacy group members
C	Executing	Carrying out or accomplishing the implementation according to plan	Carrying out or accomplishing a trial according to plan
D	Reflecting and evaluating	Quantitative and qualitative feedback about the progress and quality of implementation accompanied with regular personal and team debriefing about progress and experience	Extent to which enrollment audits and feedback/reports, trial status reports, data safety monitoring board feedback are evaluated and reflected on by trial leaders and teams

### Intervention characteristics

The “intervention characteristics” domain applies to both the interventions tested in the trial and the development of the trial protocol itself. The tested intervention, such as an experimental drug, has accompanying characteristics such as the *evidence strength* for the drug. For example, a drug with proven efficacy in the metastatic cancer setting may be more *acceptable* as the intervention in a trial in the locally advanced cancer setting. Additionally, the trial itself is an intervention with its own characteristics potentially affecting implementation success. These factors include the selection of a comparison arm, the quality of trial materials and advertising, and how adaptable a trial protocol is for each trial site.

### Outer setting

The outer setting is highly important in exerting pressure on trial-side stakeholders. These factors include relationships with other institutions, clinical trial networks (e.g., the cancer clinical trial cooperative group SWOG), and industry groups such as pharmaceutical companies. These determinants apply on an institutional basis (e.g., institutional incentives for trial enrollment) and to individual providers (e.g., pressure to compete with peers and advance careers through international reputation).

### Inner setting

In addition to relationships between institutions, characteristics within institutions may factor heavily in the successful implementation of trials. This domain is of particular importance, as there may be more variability between institutions with respect to support of trials. It also may be easier to adapt aspects such as *organizational incentives* or *available resources* at the local level to improve the success of trials. The inner setting can be conceptualized as applying to the institution itself (e.g., an academic medical center), or for larger institutions a department within the system (e.g., the department of urology).

### Characteristics of individuals

The characteristics of trialists, their teams, and individual providers may influence trial success. These include personal characteristics (e.g., beliefs about specific interventions and enthusiasm for clinical trials) and relational aspects (e.g., identification with the organization or individual sponsoring a given trial).

The characteristics of individual potential trial participants are also highly important. Most important may be potential participants’ *beliefs about intervention* with respect to their belief about the likelihood of benefit, the merits of clinical trials, and familiarity with research. Other personal attributes, such as value placed on science, trust in the medical field and institutions, and cultural influences likely largely impact the likelihood of an individual enrolling in a trial.

### Process

The process domain may be most important to improving the success of clinical trials, as it incorporates mechanisms for improvement and consolidates important factors from other domains. Constructs from this domain are more likely to be adaptable and may be modified through tailored trial improvement strategies.

## Implementation strategies to foster trial implementation and success

Once barriers to trial success are identified, trial improvement strategies should be purposefully selected to optimize effectiveness. The Expert Recommendations for Implementing Change (ERIC) presents 73 implementation strategies that can be adapted to the clinical trial context [17]. These strategies have been linked to specific CFIR determinants, permitting the identification of potential high-yield implementation strategies for a given context [19]. Linking these strategies to determinants, and creating actionable interventions based on the strategies, has the potential to target improvement interventions to each trial’s context, as opposed to relying on generic strategies that may not address the true root causes of trial problems.

## Linking determinants, outcomes, and strategies in a process model for trials

**Fig. 2:**
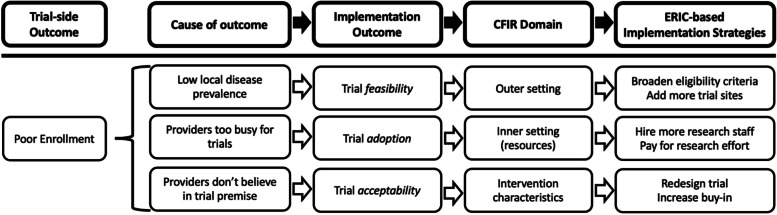
Adapted implementation research logic model (IRLM) applied to the clinical trial-side outcome of poor enrollment. *Note: arrows indicate inferential flow, not causal representations

While these frameworks have implied connections, explicitly linking them together can organize the frameworks, identify targeted mechanisms, and suggest solutions in pursuit of trial improvement. For this purpose, the final step of our worked example applies the implementation research logic model (IRLM) as a process model [14]. A process model can “provide practical guidance in the planning and execution of implementation endeavors and/or implementation strategies to facilitate implementation,” in this case supporting and framing trial improvement efforts [20]. For our application of the IRLM, we link trial outcomes with our adapted Proctor’s outcomes and CFIR determinants and identify possible implementation strategies to address these from the ERIC compilation [15–17]. Because we evaluate trial-side outcomes (analogous to clinical/patient outcomes in the original IRLM) such as poor enrollment first, we have arranged our IRLM to begin with the trial outcome, followed by the cause of this outcome (mechanism), the implementation outcome, the CFIR construct, and then a potential implementation strategy to address these barriers (Fig. 2).

Our proposed model serves to both explain the connection between the frameworks as they apply to issues with trials and makes the causal mechanism for these explicit, leading to questions that can be answered in specific targeted studies. In many ways, trial coordinating centers and site teams are constantly “solving problems” like poor enrollment and adapting to improve trial implementation. Reframing and documenting this already existing behavior more intentionally to track what worked to solve these problems (i.e., implementation strategies) and how the protocol was adapted to improve implementation outcomes (i.e., adaptation) would be enabled using our approach, promoting generalizability and broadening clinical trial practice. To demonstrate how this model may be applied, we consider worked examples of trial assessment and improvement.

## Sample use cases

### Designing a trial for successful implementation

First, the implementation of a trial should be considered while a trial is designed and the protocol is written. In a hypothetical trial for a new cancer drug, for example, the *acceptability* to providers and potential participants of both the new drug and the comparison (including the *relative advantage* of the new drug in terms of expected efficacy and toxicity) could be formally assessed, for example through surveys, interviews, or focus groups. This could both help predict *adoption* of the trial by providers and *penetration* to patients and may also directly increase participation in trials by improving the perception of involvement by both providers and patients in the design of the trial (i.e., the CFIR’s *intervention source* construct).

The *acceptability* of logistical components could also be considered. Limiting the number of visits (e.g., for lab draws or additional imaging) may improve the recruitment (i.e., *penetration*) and retention (i.e., *fidelity*) to trials, but how this affects the ability to assess endpoints and estimate efficacy of interventions (i.e., *appropriateness*) must be considered. While some of these issues may be indirectly addressed in trial design already, explicitly considering these concepts allows them to be measured and evaluated so ideal tradeoffs for different contexts can be developed.

### Struggling enrollment

Next, we consider a prostate cancer trial suffering from low enrollment. This low enrollment could be due to multiple root causes (Fig. 2). For our hypothetical trial, say we query our trial records and find only 2 out of 10 oncologists are enrolling patients onto trials (i.e., low *adoption* by providers). We conduct a survey or interviews and find providers are largely unaware of existing trials at our institution, and how many of their patients are eligible for these trials, indicating an issue of *reflecting and evaluating*. These providers may also be sensitive to *peer pressure* in comparing to their peers within the institution or at other sites. A reasonable ERIC strategy in this case may be to *audit and provide feedback*, perhaps by sending monthly emails to oncologists detailing how many participants they enrolled in trials as well as their peers’ performance. We could then evaluate how many providers are offering the trials (*adoption*) and the proportion of eligible patients enrolling (*penetration*) after the rollout of audit and feedback.

In this example, we identified and targeted the root cause of poor enrollment. If we identified other issues, we would likely have selected other strategies. An important first step, for example, would be to assess if our trial enrollment goal is *feasible*. If there are few cases of prostate cancer in the area (i.e., a CFIR *outer setting* barrier reflecting low *feasibility*), we could consider broadening eligibility criteria (ERIC strategy of *promote adaptability*) or adding more trial sites (*change service sites*). Alternatively, if provider *adoption* is high but *penetration* is low due to difficulties identifying eligible patients (CFIR *Process: Executing*), developing an electronic medical record patient screening system (ERIC: *facilitate relay of clinical data to providers* and *remind clinicians*) or hiring staff to help with trial pre-screening (ERIC: *create new clinical teams*) may be better suited interventions to improve enrollment.

The deliberate directionality of assessing root causes first is key to optimizing the successful design and targeting of interventions. If we started by designing an intervention without assessing determinants, for example, one targeted at increasing *acceptability* to patients (e.g., patient-designed information brochures), we would not be directly addressing the root cause of low provider *adoption*. As a result, we may not be optimizing trial enrollment and may not see a maximal return on investment.

### Implications for trial improvement research

This potential mistargeting may also explain why some prior trial improvement interventions may seem ineffective: they may not be asking the right questions or solving the right problems. Mistargeting an improvement intervention for a single trial may result in wasted resources, but when developing generalizable interventions for trial improvement, this mistargeting may bias the estimates of trial improvement efficacy towards the null, inappropriately suggesting interventions are ineffective when really they just are not addressing the right problems.

For example, consider a randomized study-within-a-trial (SWAT) where trial sites are randomized to receive supplemental research staff or usual research staff, aiming to increase trial enrollment [21]. Such a study may show no beneficial effect of hiring additional study staff, suggesting that hiring staff is not effective at improving enrollment. However, this may be because some of the trial sites may have already reached full *penetration*, i.e., the trial sites may already reach a high proportion of eligible patients. If this trial included only trial sites with low *adoption*, especially if this is due to few *available resources* at trial sites, more trial staff may be highly beneficial. However, because these contextual elements are not assessed, the transferability of these study findings is limited. By specifying the characteristics of trial sites and “diagnosing” determinants of trial success, we can design and evaluate trial improvement interventions for various contexts to maximize value.

In addition to informing quality improvement and prospective trial improvement studies, our worked example and proposed model can also serve as a roadmap for data-driven health services research relevant to trials. For example, understanding the interplay between cancer incidence and trial availability can inform projections of trial *feasibility* through the *outer setting* for prostate cancer trials. This approach could both inform the *feasibility* of opening a trial at a given site and highlight areas that may be scientifically underserved (i.e., with a high disease burden but few trials) where trials may thrive [22].

### Practical application of the frameworks

Applying these frameworks to diagnose trial problems and design improvements will likely require a multi-pronged approach. Context-specific determinants of trial success could be assessed through a mix of quantitative and qualitative assessment of trial site characteristics, staffing, regional and national policy, investigator, and patient characteristics tailored to specific contexts. For example, assessing *acceptability* or *relative priority* of a trial intervention may be best explored through interviews with patients and providers. Assessing trial *adoption* and *penetration* would be better assessed using trial management software and facility medical records. Many components of the *process* domain would likely require interviews with trialists and trial staff and direct observation of the trial setting, including the planning and enrollment phases. Some assessments of trial determinants may be limited by the generally poor granularity of historical trial data, rapid turnover of trial staff, and the high time and resource cost of developing and administering interviews and surveys to trial staff and patients. Developing methods to apply these frameworks to trials efficiently and expeditiously could allow for streamlined assessment and rapid cycle improvement of trial conduct.

## Value to implementation science

Considering clinical trials in the context of implementation science could both improve trials and advance the science of implementation. In addition to providing concrete examples of abstract framework constructs as noted above, the clinical trial context has components and characteristics serving as a real-world laboratory for implementation research.

First, clinical trials have multiple modifiable levels suitable for implementation interventions that may be altered more easily than other contexts. Clinical trials already require involvement and review at micro (patient-provider trial review), meso (e.g., institutional review board), and macro (e.g., national trial cooperative group) levels, providing opportunities for changes and comparisons among and between these levels. For example, implementation scientists studying adaptation could compare different sites implementing the same clinical trial [23]. Modifications could be made and evaluated at multiple levels with relative ease to compare strategies targeted at different levels, for example altering macro factors (e.g., specifying implementation factors in a trial protocol) versus micro-level factors (e.g., comparing two methods of identifying potential trial participants).

Additionally, clinical trials can support efficient implementation research through existing processes, quantity of trials, and speed of outcome generation. Since the same trial protocol (evidence-based intervention) is being implemented at each site, departures from protocols (fidelity) are already documented for data safety monitoring boards, and a key endpoint (enrollment) is already recorded; testing implementation in trials may be more efficient than in other contexts. Trial protocols already incorporate differences from trial to trial, expert trial staff are employed at many trial sites, and modifications to trial processes are expected, making targeted and measured variation in trial implementation a logical next step. Further, iterative design for implementation is made easier by the number of trials. There are thousands of trials opened annually, with over 4700 trials registered on ClinicalTrials.gov opening after January 1, 2022, in oncology alone. Strategies developed as one trial is launched could be incorporated into upcoming trials, with an array of characteristics and settings to choose from resulting in rich inference for implementation research. The speed of some trial endpoints and outcomes also could permit rapid inference and iteration for implementation research. For example, trial enrollment is a continuously recorded leading indicator of trial success. Outcomes like adoption and penetration could be measured continuously while trial implementation studies are conducted, also allowing for advanced implementation trial designs with potentially enhanced inference and efficiency (e.g., crossover, sequential multiple assignment randomized trials (SMART)) [24].

In all, the clinical trial context could likely benefit from implementation science approaches, but also has great potential as an efficient laboratory for implementation research. These refined approaches and frameworks could then be transferred to other evidence-based practice settings.

## Conclusion

Clinical trials are complex interventions with evidence-based benefits but frequently suffer from poor implementation. Adapting implementation science frameworks to the clinical trial context can foster shared vocabulary improving the design, implementation, testing, science, and practice of clinical trials. A consolidated, systematic, logical approach to clinical trial improvement appears warranted to address return on investment concerns for the clinical trials enterprise and deliver on the promises of advancing science, patient care, and fostering public health.

## Data Availability

Not applicable.
