# Correction: Applying implementation frameworks to the clinical trial context

**DOI:** 10.1186/s43058-023-00413-7

**Published:** 2023-03-14

**Authors:** Kristian D. Stensland, Anne E. Sales, Laura J. Damschroder, Ted A. Skolarus

**Affiliations:** 1 Department of Urology, Dow Division of Health Services Research, University of Michigan, NCRC Building 16, 100S-12, 2800 Plymouth Road, Ann Arbor, MI 48109 USA; 2 Department of Learning Health Sciences, University of Michigan, Ann Arbor, MI USA; 3 Center for Clinical Management Research, VA Ann Arbor Healthcare System, Ann Arbor, MI USA; 4 Sinclair School of Nursing, University of Missouri, Columbia, MO USA; 5 Department of Family and Community Medicine, University of Missouri School of Medicine, Columbia, MO USA

**Correction: Implement Sci Commun 3, 109 (2022)**

**https://doi.org/10.1186/s43058-022-00355-6**

Following the publication of the original article [1] the authors requested to update the “Competing interests” section as follows:

“The authors declare that Anne Sales is co-Editor-in-Chief of the journal."
